# Pre-extensively Drug-Resistant Pulmonary Tuberculosis With a Giant Primary Splenic Hydatid Cyst With Multiple Adverse Drug Reactions

**DOI:** 10.7759/cureus.55722

**Published:** 2024-03-07

**Authors:** Sankalp Yadav

**Affiliations:** 1 Medicine, Shri Madan Lal Khurana Chest Clinic, New Delhi, IND

**Keywords:** mtb (mycobacterium tuberculosis), cbnaat/ xpert/ rif assay, pre-extensively drug resistant pulmonary tuberculosis, echinococcus, primary splenic hydatidosis, giant splenic cyst, spleen

## Abstract

Drug-resistant tuberculosis is a noteworthy threat to public health, especially in high-burden countries. Management of these types of tuberculosis is lengthy and associated with a number of adverse drug reactions. Pre-extensively drug-resistant tuberculosis is a serious type of disease that is caused by the strains of *Mycobacterium tuberculosis* that are resistant to either rifampicin or both, i.e., rifampicin and isoniazid, and resistant to any fluoroquinolones. A splenic hydatid cyst is relatively rare and has never been reported in a case of pre-extensively drug-resistant pulmonary tuberculosis. The present case is a rare case of a young Indian male who was diagnosed with pre-extensively drug-resistant pulmonary tuberculosis through a cartridge-based nucleic acid amplification test and second-line drug susceptibility testing. Further, a diagnostic radiometric investigation showed a giant hydatid cyst in the spleen. He was started on an all-oral longer treatment regimen per the existing protocols. However, his treatment was associated with multiple adverse drug reactions.

## Introduction

In high-burden countries like India, public health is at risk because of infectious diseases like tuberculosis [1]. In recent years, there has been a significant increase in the number of tuberculosis cases. This could be attributed to a more streamlined notification and monitoring of the program. Nonetheless, the incidence and prevalence of tuberculosis remain at 188 and 312 per 0.1 million people, respectively, even with such vigilante measures [2].

A significant challenge with tuberculosis control is drug-resistant tuberculosis. Recent years have witnessed an increase in the overall number of drug-resistant tuberculosis cases [3]. Drug-resistant tuberculosis falls into four groups: isoniazid-resistant tuberculosis, pre-extensively drug-resistant tuberculosis, multi-drug-resistant/rifampicin-resistant tuberculosis, and extensively drug-resistant tuberculosis [4].

Pre-extensively drug-resistant tuberculosis is now defined as tuberculosis caused by strains of *Mycobacterium tuberculosis* that meet the criteria for both multidrug-resistant and rifampicin-resistant tuberculosis, as well as resistance to any fluoroquinolone [5]. Per the India TB Report 2023, about 2,411 cases out of 10,143 samples were of pre-extensively drug-resistant tuberculosis. Besides, about 93% of these cases were initiated on treatment [4].

A splenic hydatid cyst, a result of infection by *Echinococcus granulosus *larvae, is an infrequent condition, with less than 3% of cases reported even in endemic countries. It is often associated with vague symptoms, which makes its diagnosis challenging [6].

The simultaneous occurrence of a splenic hydatid cyst with pre-extensively drug-resistant pulmonary tuberculosis makes it difficult to diagnose and manage the situation. Herein, the case of a 41-year-old Indian male is described. He presented with complaints of fever, cough, abdominal pain, and loss of appetite. A diagnosis of pre-extensively drug-resistant pulmonary tuberculosis with a splenic hydatid cyst was made, and treatment was initiated.

## Case presentation

A 41-year-old Indian man from a low socioeconomic background reported fever, cough with expectoration, abdominal pain, and appetite loss in 2022. He was asymptomatic two weeks prior, and then he had a low-grade evening-rise fever without chills or rigors. Further, he had a cough with yellow-colored expectoration for one week, which was continuous, non-blood-tinged, and not associated with any aggravating or relieving factors. He also reported intermittent left-sided abdominal pain that was insidious in onset for two weeks. Furthermore, there was a loss of appetite; however, there was no significant weight loss.

No prior history of trauma, convulsions, or unusual weight loss was present. He was a delivery boy with a history of working on farms. Also, he had a history of tuberculosis twice in the past (the first episode of drug-sensitive pulmonary tuberculosis in 2002 with a treatment outcome as cured and the second in 2008, which was also drug-sensitive pulmonary tuberculosis with a treatment outcome as cured). There was no record of migration, abuse of drugs, imprisonment, camp stays for refugees, or night shelter stays. Furthermore, there was no prior surgical history.

A general examination revealed a 98.6 °F temperature, 79 beats per minute pulse, 110/80 mmHg blood pressure, 17 breaths per minute respiratory rate, 68 kg weight, and 99% oxygen saturation (SpO_2_) on room air. His systemic examination was remarkable for bilateral crackles on auscultation. Additionally, the abdominal examination was remarkable for tenderness in the left hypochondrium; however, no visceromegaly was noted on palpation. Moreover, clubbing, icterus, cyanosis, pallor, koilonychia, and lymphadenopathy were absent.

He underwent standard radiographic and blood investigations. The sputum fluorescent microscopy was positive (2+) for *Mycobacterium tuberculosis*. This was further confirmed as drug-resistant tuberculosis with low detection of *Mycobacterium tuberculosis* with resistance to rifampicin on a cartridge-based nucleic acid amplification test (CBNAAT).

**Figure 1:**
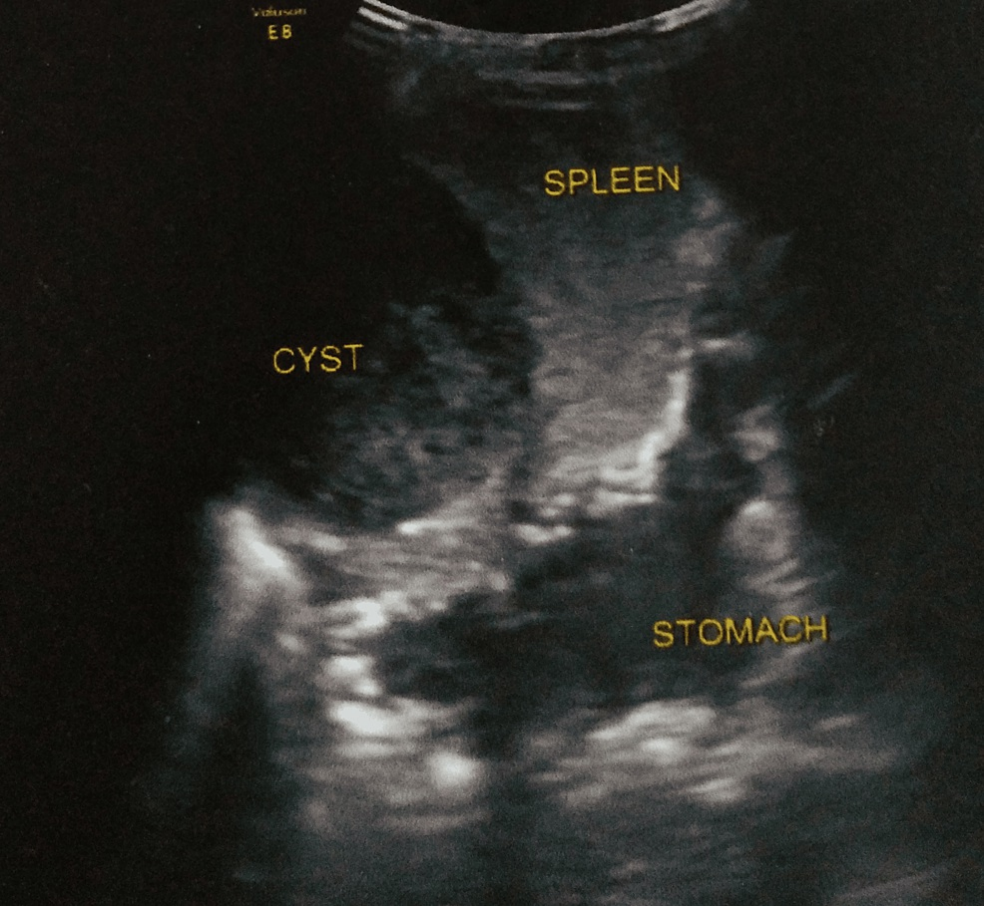
USG of the abdomen showing a well-defined cystic lesion USG: ultrasonography

Ultrasonography of the abdomen revealed a well-defined, fairly thick-walled cystic lesion measuring 66 x 73 x 59 cm in the spleen. It showed coarse echoes and curvilinear calcifications in the wall (Figure 1).

**Figure 2:**
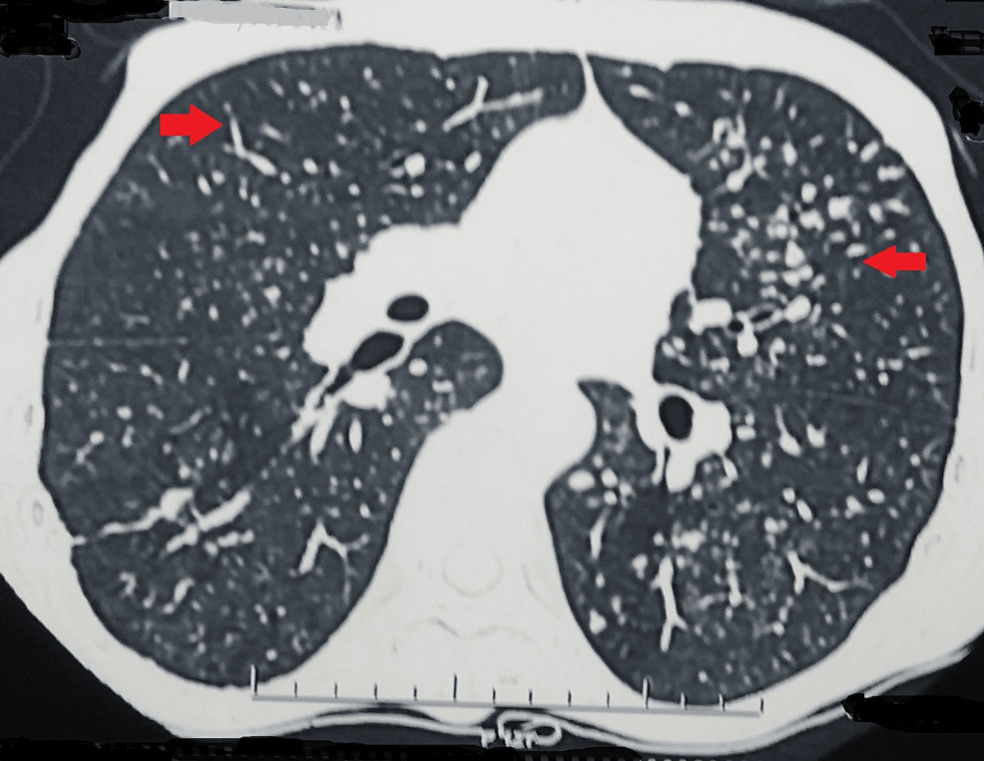
Computed tomography pulmonary angiogram showing bilateral involvement of the lungs

Further, the patient complained of hemoptysis (10-15 ml per episode); hence, a computed tomography pulmonary angiogram was performed, which was suggestive of multiple centrilobular nodules of varying sizes scattered in bilateral lung parenchyma, with many of them showing a tree-in-bud appearance (Figure 2).

**Figure 3:**
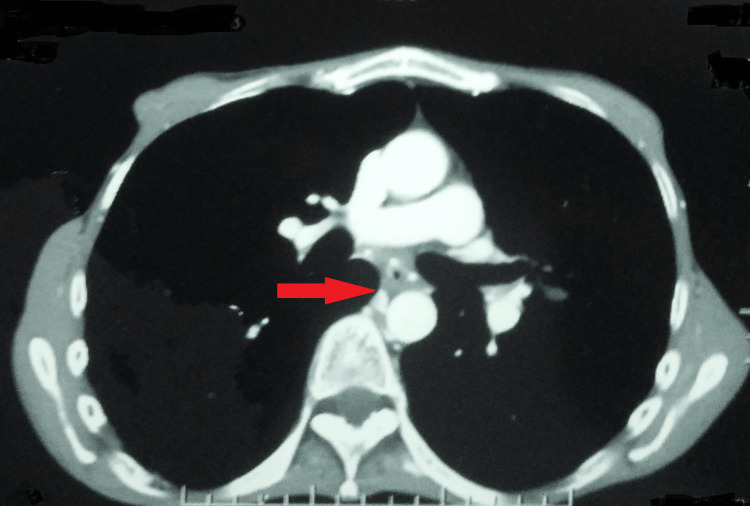
Computed tomography pulmonary angiogram suggestive of hypertrophied and tortuous bronchial artery

**Figure 4:**
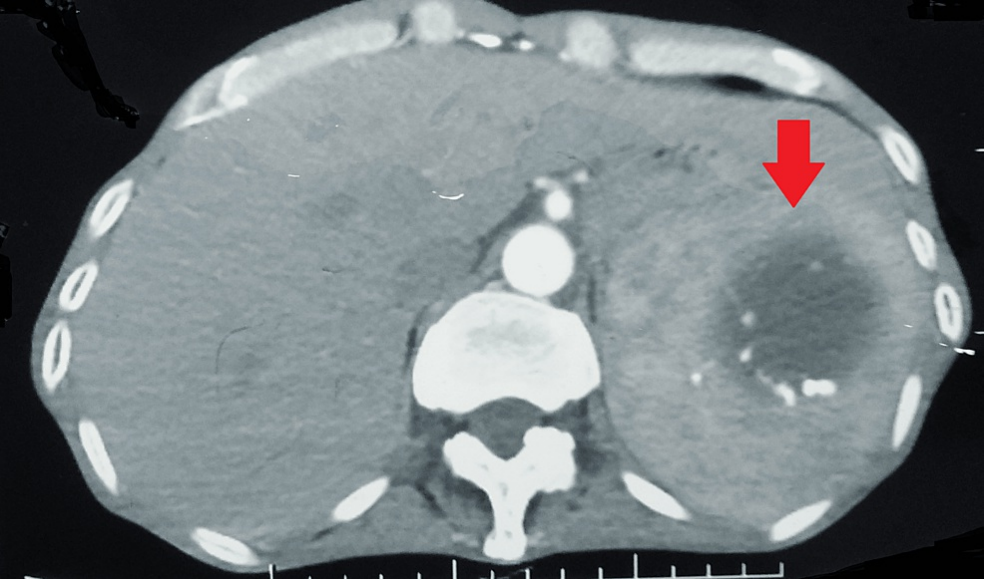
Computed tomography of the lungs showing a well-defined fluid-attenuating cystic lesion with peripheral wall calcifications in the spleen

There were hypertrophied and tortuous common bronchial, 5th, 6th, and 7th intercostal arteries. A well-defined fluid-attenuating cystic lesion with peripheral wall calcifications was seen in the spleen, likely a hydatid cyst (Figures 3, 4).

Meanwhile, reports of second-line drug-susceptibility testing were suggestive of fluoroquinolone resistance (low). Hence, a final diagnosis of pre-extensively drug-resistant pulmonary tuberculosis with a primary splenic hydatid cyst was made, and he was advised a pre-treatment evaluation (Table 1).

**Table 1:** Pre-treatment evaluation ELISA: enzyme-linked immunosorbent assay; IgG: immunoglobulin G; QTcF: corrected QT interval by Fredericia

Test	Result	Reference range
Hemoglobin	9.9 g/dL	11.9-15
Platelet count	1.9 x 10^9^/L	1.5-4.0 x 10^9^
Total leukocyte count	7.4 × 10^9^/L	4-10
Erythrocyte sedimentation rate	63 mm/hr	0-20
Bilirubin (conjugated)	0.9 µmol/L	<1
Human immunodeficiency virus	Non-reactive	Reactive-Non-reactive
Fasting blood sugar	4.20 mmol/L	3.9-5.6
Serum electrolytes (Sodium, Potassium, Magnesium, and Calcium)	Normal	Normal-Abnormal
Serum creatinine	55 µmol/L	53-97.2
Urine routine and microscopic	Normal	Normal-Abnormal
Serum thyroid-stimulating hormone levels	0.5 mU/L	0.4-4.0
Serum uric acid	3.7	3.5-7.2 mg/dL
Electrocardiogram	Normal (baseline QTcF-387 ms)	Normal-Abnormal
ELISA for detection of IgG antibodies for* Echinococcus*	Positive	Positive-Negative

As his pre-treatment evaluation was within the reference range, a weight-based all-oral longer regimen per the national guidelines was initiated (Table 2).

**Table 2:** An all-oral longer regimen per the national guidelines PO: per oral; OD: once a day; AD: alternate day; BD: twice a day

Drug	Route of administration	Dose	Duration
Bedaquiline	PO	400 mg X OD and then 200 mg AD	24 weeks (2+22)
Linezolid	PO	600 mg X OD, followed by 300 mg X OD	18 months (6+12) but replaced with delamanid 100 mg X BD at the sixth month
High-dose moxifloxacin	PO	600 mg X OD	540 days
Clofazimine	PO	100 mg X OD	540 days
Cycloserine	PO	500 mg X OD	540 days
Pyridoxine	PO	100 mg X OD	540 days

For the splenic hydatid cyst, he was referred to the surgeon who advised a splenectomy but the patient refused the same. He was on medical management with tablet albendazole. A review by the nodal drug-resistant tuberculosis center stated that the splenectomy could be offered post-completion of his all-oral, longer treatment.

He continued his treatment with occasional episodes of adverse drug reactions like a raise in corrected QT interval by Fredericia (QTcF) (519 ms), which was promptly managed with a correction of the electrolytes. He also had linezolid-induced grade III paresthesia detected on the nerve conduction study, which was managed with the replacement of linezolid with delamanid at the sixth month.

**Figure 5:**
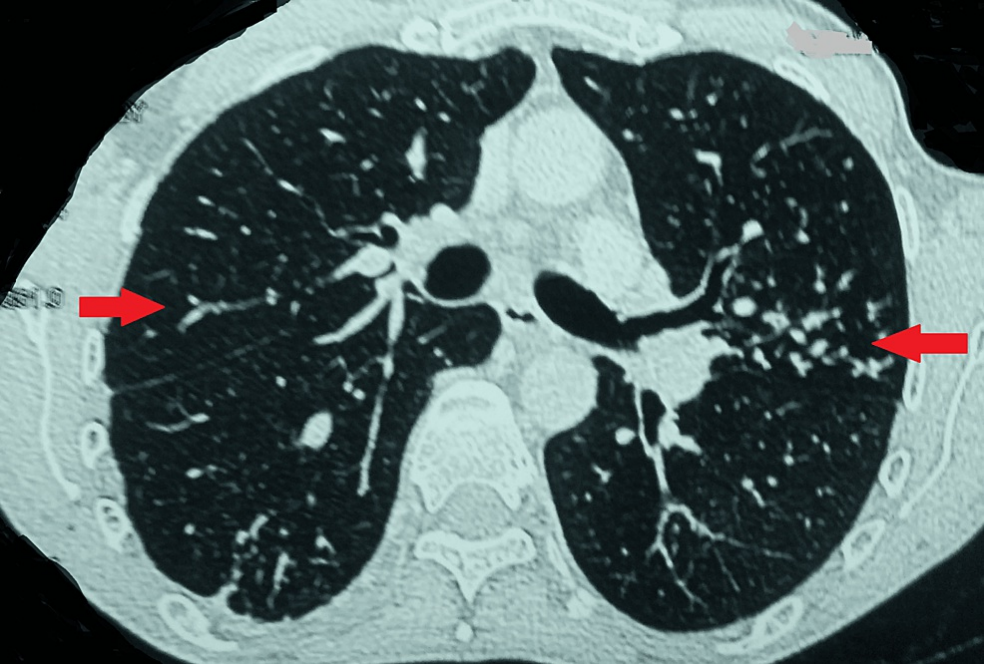
A follow-up contrast-enhanced computed tomography of the chest suggestive of acute-on-chronic infective sequelae

**Figure 6:**
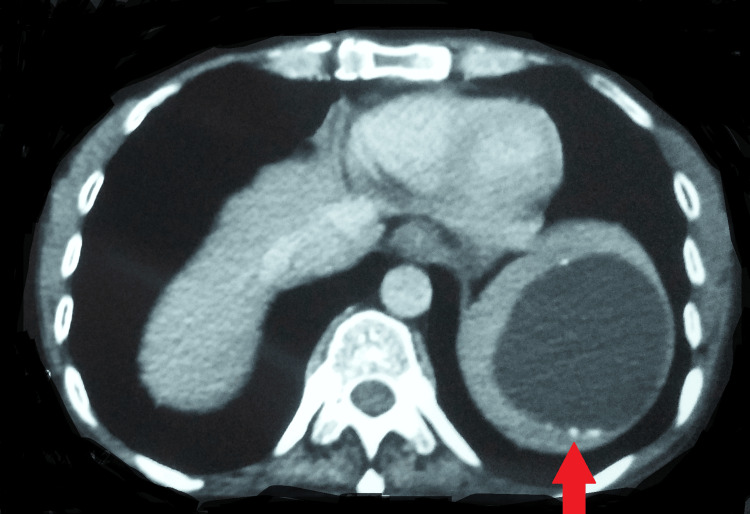
Contrast-enhanced computed tomography showing a well-defined cyst in the spleen

A follow-up contrast-enhanced computed tomography of the chest at the completion of 18 months was suggestive of acute-on-chronic infective sequelae and revealed multiple discrete centrilobular nodules scattered in the bilateral lung parenchyma (left > right) many of them showing a tree-in-bud appearance. Multiple fibrocalcific nodules and fibroatelactic changes were also seen in the bilateral upper lobes. Note was made of a large hypodense cystic lesion of size 6.3 x 6.4 cm seen at the upper pole of the spleen, showing peripheral calcific foci, likely a hydatid cyst (Figures 5, 6).

**Figure 7:**
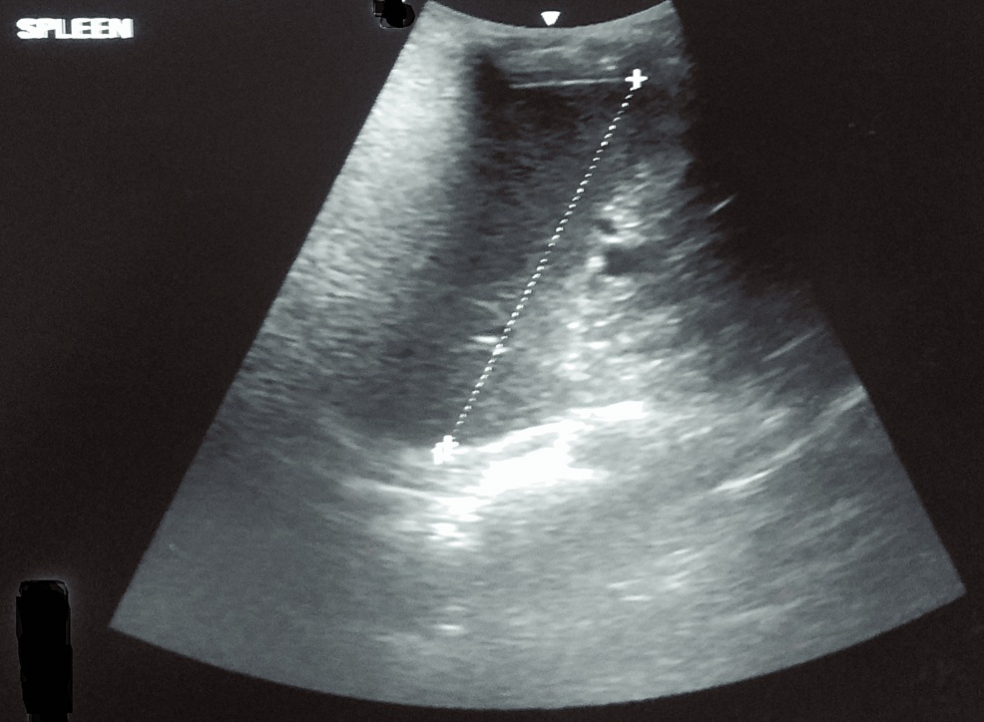
Latest ultrasonography showing a well-defined cystic lesion in the spleen

As a result, his treatment was extended for two more months. Presently, he is on extended treatment with no fresh complaints. However, even after regular counseling, he is reluctant to go for surgical management of the hydatid cyst. He was monitored with regular ultrasonograms for the splenic cyst and the latest reports show a well-defined cystic lesion in the spleen (Figure 7).

## Discussion

Drug-resistant tuberculosis is a big problem in achieving the goals of tuberculosis elimination in countries like India. It is widely reported that drug resistance is an outcome of man-made factors such as inadequate drug therapy, ignorance on the part of the prescribing physician or surgeon, difficulty acquiring drugs for low-income patients because of fiscal issues or social security, frequent shortages of second-line antituberculous drugs because of poor management and/or financial constraints, use of drugs or fixed drug combinations that contain unknown bioavailability of the drugs, lack of motivation at the beginning of treatment, and insufficient self-administration of drugs not being closely monitored during the intensive phase of therapy [7].

Diagnosis of pre-extensively drug-resistant tuberculosis is a challenging task, mainly due to the delay in the reporting of the patients at the health facilities. The National Tuberculosis Elimination Program of India is targeted at tuberculosis elimination, and the same is evident from the timely detection and initiation of management in the present case [8]. However, the treatment is lengthy and costly (in the private sector with exceptions), associated with a high pill burden, social stigma, adverse drug reactions, and a complex process that calls for the knowledge and abilities of a team that includes a counselor, a pulmonologist, and an expert in infectious diseases [3]. The primary approach is conservative, with management by second-line antituberculous medicines. In India, the World Health Organization recommends an all-oral-longer therapy [9]. But this management is associated with a large number of adverse drug reactions that are often asymptomatic, as seen in the present case, like the raised QTcF that was noted at the routine follow-up and the grade-III paraesthesia that was managed with the replacement of linezolid.

The prevalence of a splenic hydatid, which is the third most typical location for *Echinococcus*, is 0.5-4% worldwide. Iran has the highest incidence (4%). Hydatid cysts are nearly the only type of parasitic splenic cysts, with about 50-80% of splenic cysts in endemic locations being echinococcal. Reports of splenic hydatidosis date back thousands of years [10]. The first person to report splenic hydatidosis as an autopsy finding was Berlot in 1790. A splenic hydatid cyst could be either primary (isolated to the spleen only) or secondary (accompanied by hydatid cysts in other organs). Primary splenic hydatid cysts are extremely rare and reported in less than 2% of patients [11]. It could be detected accidentally or manifest as vague symptoms. It is native to countries in South America, Africa, the Middle East, South Europe, India, and Australia that raise cattle. The management is mainly surgical, with splenectomy [10]. Large cysts can rupture, causing life-threatening conditions like abdominal pain, urticaria, anaphylaxis, and sudden death [12]. However, in asymptomatic cases or where symptoms are not remarkable, convincing patients for surgery is an arduous task.

Cases of hydatid cysts of the lung with tuberculosis are reported in the literature, but a detailed literature search revealed that a primary splenic hydatid cyst in a pre-extensively drug-resistant pulmonary tuberculosis case is never reported.

Although this is only one such report, it is essential that clinicians report such rare presentations, especially from high-burden countries. This will not only help in knowledge dissemination but will also aid in making or modifying the existing treatment protocols.

## Conclusions

A case of pre-extensively drug-resistant pulmonary tuberculosis with a splenic hydatid cyst has not been reported before to the best of this author's knowledge.

This case emphasizes the need for a detailed workup, as was done in this case, where the hydatid cyst was diagnosed promptly. However, this case also highlighted the importance of counseling and monitoring for adverse drug reactions, which could be asymptomatic at times. Additionally, cases with such rare presentations are infrequently seen even in endemic countries, which stresses periodic training and dissemination of information about these cases so that fatal outcomes due to diagnostic delays can be avoided.
